# Fine-Scale Mapping by Spatial Risk Distribution Modeling for Regional Malaria Endemicity and Its Implications under the Low-to-Moderate Transmission Setting in Western Cambodia

**DOI:** 10.1371/journal.pone.0158737

**Published:** 2016-07-14

**Authors:** Suguru Okami, Naohiko Kohtake

**Affiliations:** Graduate School of System Design and Management, Keio University, Kanagawa, Japan; Centro de Pesquisa Rene Rachou/Fundação Oswaldo Cruz (Fiocruz-Minas), BRAZIL

## Abstract

The disease burden of malaria has decreased as malaria elimination efforts progress. The mapping approach that uses spatial risk distribution modeling needs some adjustment and reinvestigation in accordance with situational changes. Here we applied a mathematical modeling approach for standardized morbidity ratio (SMR) calculated by annual parasite incidence using routinely aggregated surveillance reports, environmental data such as remote sensing data, and non-environmental anthropogenic data to create fine-scale spatial risk distribution maps of western Cambodia. Furthermore, we incorporated a combination of containment status indicators into the model to demonstrate spatial heterogeneities of the relationship between containment status and risks. The explanatory model was fitted to estimate the SMR of each area (adjusted Pearson correlation coefficient R^2^ = 0.774; Akaike information criterion AIC = 149.423). A Bayesian modeling framework was applied to estimate the uncertainty of the model and cross-scale predictions. Fine-scale maps were created by the spatial interpolation of estimated SMRs at each village. Compared with geocoded case data, corresponding predicted values showed conformity [Spearman’s rank correlation r = 0.662 in the inverse distance weighed interpolation and 0.645 in ordinal kriging (95% confidence intervals of 0.414–0.827 and 0.368–0.813, respectively), Welch’s t-test; Not significant]. The proposed approach successfully explained regional malaria risks and fine-scale risk maps were created under low-to-moderate malaria transmission settings where reinvestigations of existing risk modeling approaches were needed. Moreover, different representations of simulated outcomes of containment status indicators for respective areas provided useful insights for tailored interventional planning, considering regional malaria endemicity.

## Introduction

### Remaining issues and emerging challenges toward malaria elimination

Despite the many efforts over many years to contain and then eliminate malaria, Malaria remains an important global health threat that still causes hundreds of thousands of deaths every year [1]. Substantial efforts toward malaria containment by many stakeholders have decreased the burden of this infectious disease in a number of endemic regions. Now, decades after the global malaria eradication program, malaria elimination again features on the global health agenda [2]. In recent years, an increasing number of countries with low-to-moderate transmission areas have implemented actions to eliminate malaria from their entire territories [3]. In Cambodia, the target is to be malaria free by 2025 [4]. Recent activities have decreased the incidence of malaria in Cambodia to less than half the incidence in the early 2000s [5]. Currently, about half the Cambodian population are living in malaria-free or low-transmission settings [6]. However, a number of issues remain and new challenges are emerging in the efforts to eliminate malaria. The emergence of artemisinin resistance, which has been reported mostly in the Greater-Mekong subregions, is one of the new challenges [7]. Artemisinin is a potent and rapidly acting blood schizonticide that is effective for all plasmodium species [8]. No alternative effective antimalarial treatment is available at present; therefore, the consequence could be dire if resistance spreads to wide geographical regions [9]. A number of reports have emerged of delayed parasite clearance in patients in western Cambodia taking artemisinin [10–13]. One recent report showed that the artemisinin-resistant malaria parasite had the potential to infect vectors in other geographical regions [14]. The reported treatment failures in western Cambodia varied depending on the conditions [11, 15–17]; however, all the reports strongly emphasized the urgent need to address this issue. Appropriate medication is undoubtedly important and in areas such as those close to western Cambodian border, this approach occasionally needs intensive care and monitoring of patients. To attain the desired outcomes, several studies such as those focused on screening and treatment [18], community-based surveillance [19], and mass drug administration [20] have been piloted. What is common to these interventions was the recognition that intensive support and monitoring for local practitioners were critical in obtaining the desired outcomes. Healthcare resources cannot be used inexhaustibly; therefore, identification of the target hotspots in malaria endemic areas, delivery of sufficient stockpiles of resources, and intimate support for local healthcare providers are essential, especially in remote endemic regions where accessibility cannot be retained over a whole year.

### Limitations of malaria risk modeling and mapping approaches

Recent efforts to quantify the risk burden and the creation of spatial prediction maps of malaria risk have made substantial contributions toward identifying target hotspots [21–22]. A world map of *Plasmodium falciparum* malaria endemicity has been published using a parasite rate surveillance report and a model-based geostatistical approach [23–24]. Remote sensing techniques are powerful tools that can be used to identify hotspots and to investigate malaria epidemiology [25]. Several environment-related indices calculated from remote sensing data, such as normalized difference vegetation index, normalized difference water index, and topological wetness index have been used to predict regional malaria endemicity [26–29]. Climate also is closely related to the risk of malaria [30–31]. Cohen et al. [32] created fine-scale risk maps of both high endemic and low endemic seasons in Swaziland from routinely collected individual case data combined with environment-related indices calculated from remote sensing data. Despite these advances, the current decrease in the prevalence of malaria causes the changes in its data availability and case measurement, which indicates that the risk mapping approach needs to be adjusted to take this new situation into account. Under low malaria transmission settings where few infectious cases are reported, the sample size required to both estimate and spatially predict infection prevalence becomes very large, and such information usually cannot be obtained on a fine scale. Instead, cross-scale predictions using data collected on a coarser scale can be performed using a Bayesian modeling framework [33]. To measure the malaria disease burden when malaria becomes rare, it becomes increasingly difficult to detect ongoing transmission monitoring by parasite rate [34]. Furthermore, an intensive focused screening method indicated that, in low-transmission settings, many malaria cases were asymptomatic, which made it difficult to identify all the cases by passive surveillance systems [18, 35]. Because situations like this are important steps toward malaria elimination, there is an urgent need to reinvestigate the modeling method of disease burden under low-to-moderate transmission settings while establishing the strong surveillance system. Under the conditions that are supported by rigorous surveillance systems, annual parasite incidence (API) can be a reliable measure for reporting new malaria infections under a low-to-moderate transmission situation [36]. Here, we applied a mathematical modeling approach for standardized morbidity ratio (SMR) calculated by API using routinely aggregated surveillance reports and variables related to human interactions with surrounding environmental conditions to create fine-scale spatial risk distribution maps of two provinces, Pailin and Preah Vihear, in western Cambodia. Under the ever-changing local endemic conditions, all interventions need be reviewed carefully and tailored for regional circumstances in an ongoing way to ensure that they remain fully effective. The progresses of malaria containment actions are expected to affect these conditions. We also incorporated the combination of two containment status indicators into the model to demonstrate spatial heterogeneities of the relationship between containment status and risks to support informed decision-making for more efficient resource allocations and intervention planning, considering spatial descriptions of regional malaria endemicity.

## Materials and Methods

### Malaria data collection

Malaria case data were collected from the Cambodia Malaria Bulletin report from 2010 to 2013 [37–38]. This dataset was built from case reports collected through the efforts of the Malaria Information System and the national facility-based Health Information System using a common coding system [39]. It contains the API (per 1000 people) in each health operational district for two malaria species, *P*. *falciparum* and *P*. *vivax*, reported by healthcare facilities or village malaria workers. The SMR, standardized mortality or morbidity ratio, is expressed as a ratio or percentage of quantifications compared with the general population of interest (Eqs 1 and 2) [40].

$$SMR={\hat{\theta }}_{\;i}=\frac{{ο}_{i}}{e_{i}}$$ (1)

$$e_{i}=\underset{k}{\sum }n_{ik}\;P_{k}$$ (2)

where, *o*_*i*_ is the observed number of cases in *i* area, *e*_*i*_ is the expected number of cases in *i* area, *n*_*ik*_ is the population in *k* age group in *i* area, and *P*_*k*_ is the incidence of clinical cases in *k* age group in the reference population. *e*_*i*_ was estimated by multiplying the population and reported incidence and aggregating them for each age group in 10 provinces in western Cambodia [41]. Since, API was reported incidence per 1,000 people, SMR, ${\hat{\theta }}_{\;i}$ in *i* district, was calculated by dividing the API by *e*_*i*_ per 1,000 people. Assuming small observed case numbers and relatively large dispersions under the low-to-moderate transmission setting, the observed case count data *o*_*i*_ can be assumed to follow the negative binomial distribution, *o*_*i*_ | *μ*_*i*_, where *μ*_*i*_ is the corresponding distribution mean and *ρ* is the scale parameter (Eq 3). Then, by transforming Eq 1, *μ*_*i*_ can be derived by multiplying *e*_*i*_ and the relative malaria risk, ${\hat{\theta }}_{\;i}$ (Eq 4) [42]. Hence, SMR can be used for estimating the case number of target area, which is also useful for informed decision-making of healthcare resource allocation.

$$o_{i}\;|\;\mu_{i}∼\text{NegBin}\;\left(\mu_{i},\rho \right)$$ (3)

$$\mu_{i}=e_{i}\;{\hat{\theta }}_{\;i}$$ (4)

Considering the small number of observed cases compared with population size under the low-to-moderate malaria transmission setting and thereby raising a concern for the modifiable areal unit problem in geographical analysis [43–44], the SMR for each health operational district was smoothed using the empirical Bayes method (EBSMR) [45] to adjust the influence of different population size in area units.

### Environmental and non-environmental anthropogenic covariates

The covariates that were incorporated into the modeling framework are described in Table 1. The normalized difference vegetation index (NDVI), the normalized difference water index (NDWI), and the land surface water index (LSWI) were calculated from Terra-MODIS 8-day composite data (http://LPDAAC.usgs.gov) from 2010 to 2013. Because EBSMR was represented as yearly average, these environmental variables were averaged to the mean values for each year.

The digital elevation model at 30-m resolution was extracted from the ASTER GDEM database (http://gdem.ersdac.jspacesystems.or.jp) [46] and used to estimate the altitude. The topographic wetness index (TWI) was calculated using this altitude model and estimated by the method described previously [47]. Considering the interactions between surrounding environment and people in the malaria transmission process, we extracted data from multiple surrounding circular buffers with different radius distances (i.e., for each 1 km from 1–5 km) from villages. Environmental covariates extracted from each village were aggregated to the district level to reflect the overall condition of target districts. As the number of villages directly relates to the aggregated value, they were taken averages by the number of villages in each health operational district. These data, which could potentially indicate human interactions with the surrounding environment, were compared by calculating the correlation and coefficient of determination for the models. Because temperature can influence the ecology of mosquito breeding habitats, and therefore malaria transmission [30], we collected the *Plasmodium* temperature suitability index [48] from the Malaria Atlas Project database [49]. Rapid urbanization is related to changes in the risk patterns of malaria transmission compared with sparsely populated rural areas [50–51], and susceptibility of these two different populations can be influenced by of the types of containment actions that are implemented. Population density per km^2^ was calculated as a variable reflecting the extent of urbanization, using records in the Cambodia Malaria Bulletin divided into the areas of each health operational district. Furthermore, we used the reported proportion of sufficient ownership of long-lasting insecticide-treated nets (LLIN_suf_) [41] and the treatment failure rate of artemisinin (TF_rate_) [52] as containment status indicators. LLIN_suf_ is defined as the proportion of households in which distributed mosquito nets cover no more than two persons per net. Because no geographical localities could be obtained for these indicators, they were aggregated to the provincial level and incorporated into the model.

**Table 1:** Variables used to build the modeling framework to estimate EBSMR.

Category	Variable	Data source	Data collection
Vegetation	NDVI	Terra-MODIS 8-day composite data 2010–2013	Extracted mean value from 1, 2, 3, 4, and 5 km surrounding circular buffer from each populated village
Water	NDWI	Ditto	Ditto
	LSWI	Ditto	Ditto
Geography	TWI	Digital elevation model at 30 m resolution from ASTER GDEM database [46]	Ditto
Temperature	*P*. *falciparum* temperature suitability index (*Pf*TSI)	Malaria Atlas Project database [49]	Averaged to mean value for each HOD
Population	Population density (/km^2^)	Cambodia Malaria Bulletin report 2010–2013 [37–38]	Population record divided by total areas of each HOD
Vector control	Sufficient ownership of LLIN^a^	Cambodia Malaria Survey 2010 [41]	Used the values reported at each provincial level
Treatment	Treatment failure rate by artemisinin combination therapy^b^	National Center for Parasitology, Entomology and Malaria Control [52]	Ditto

^a^Proportion of household in which distributed mosquito nets cover no more than two persons per net.

^b^Test positive for *P*. *falciparum* on day 28 or day 42.

EBSMR, Standardized morbidity ratio estimated by empirical Bayese method; NDVI, Normalized difference vegetation index; NDWI, Normalized difference water index; LSWI, Land surface water index; LLIN, Long-lasting insecticide-treated net; TWI, Topographical wetness index; HOD, Health operational district.

### Spatial risk distribution modeling

The relationship between EBSMR ($\hat{\theta }$) and spatial covariates was modeled using a generalized linear regression model as a function of the *N* predictive variables (*X*,*Z*), given that the logarithmic $\hat{\theta }$ follows Gaussian distribution.

$$\hat{\theta }=e^{\lambda }$$ (5)

$$\lambda =\alpha +\underset{N}{\sum }\beta_{N}{Χ}_{N}+\underset{N}{\sum }\gamma_{N}Z_{N}+\varepsilon$$ (6)

Where *α* is the model intercept, *β* is the parameter associated with environmental covariates *X* and *γ* with non-environmental anthropogenic covariates *Z*. The maximum likelihood of observed data provided to the model and the input predictors were calculated based on this modeling frame (Eqs 5 and 6). Data modeling was conducted at the district level scale. For model fitting, either maximum likelihood or Markov chain Monte Carlo (MCMC) methods can be used. We first used the maximum likelihood method to examine the predictor variables and then, based on the results, we used the MCMC method in the Bayesian modeling frame to estimate the uncertainty about the relationships represented by *α*, *β*, and *γ* (Eq 6) and cross-scale predictions. The models were fitted using the R software (https://www.r-project.org). Predictor variables were entered into the initial models in a stepwise manner to identify the variables to be incorporated into the model, and then both sets of variables were entered into the model. This approach was repeated until all remaining variables in the final model were significant at *α* = 0.05. An MCMC sampler in the JAGS framework [53] was used for the Bayesian model fitting. Three MCMC chains with 50,000 iterations as burn-in and 30,000 iterations thinned every 30 were stored as parameter estimates. Convergence of the model was examined by Gelman-Rubin diagnostics [54] and by visual assessment of the trace plots of chains.

### Mapping and validations

The fitted model was applied in conjunction with spatial covariates extracted from the location of each village to estimate the village level SMR. This process can be considered as the disaggregation process of aggregated environmental covariates once used for modeling at the district level scale. Values of estimated village level SMR were used as skeletons of the spatial interpolation. Realized values calculated by spatial interpolation methods were plotted in each 250 m x 250 m spatial grid. We created maps that visualized the risks of two provinces, Pailin and Preah Vihear, in western Cambodia by the inverse distance weighed (IDW) method and ordinal kriging interpolation of the estimated SMR at each village. To evaluate the accuracy of the cross-scale prediction from the model, the predicted SMR was compared with geocoded case data for Pailin [55] and Preah Vihear [56] collected from the Malaria Atlas Project database [48] using Spearman’s rank correlation [57] and Welch’s t-tests for unequal variances [58]. The source data of our maps were mostly from the reports from the village malaria workers and the Health Information System and were based on the rapid diagnostic kit (RDT) and microscopy detection. These data were selected because of the detection methods (RDT/microscopy) used and were closest to the reported period from the study period. To exclude the incidental nature for Spearman’s correlation with this sample data, we resampled the dataset 2,000 times with replacement to create confidence intervals with the non-parametric bias corrected and accelerated percentile method [59] to assess the distribution of correlation values. Since, we aimed to provide useful information to the practitioners, visual representations of risk distributions in the maps were also validated for their agreement with those in existing risk maps and utilities of the maps for deciding target areas through interviews with health care providers in the regional health center and with geographical information system professionals.

## Results

**Fig 1:**
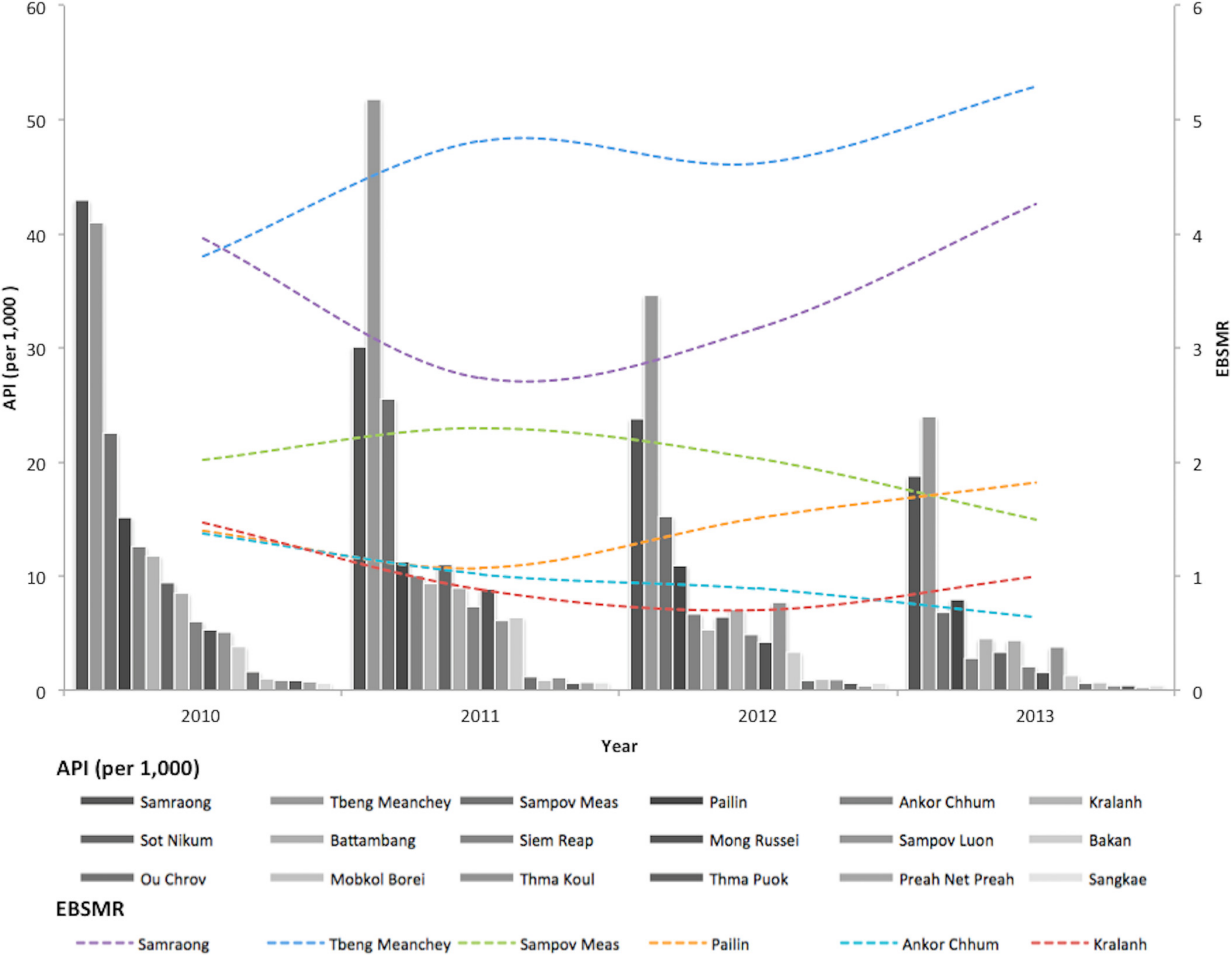
Annual parasite incidence (API) of western-Cambodian health operational districts and empirical Bayese estimated standardized morbidity ratio (EBSMR) for 6 operational health districts with high EBSMR ^a^. Bar graph represents API in each health operational district and dotted line represents EBSMR of 6 provinces with high EBSMR. ^a^ District with higher EBSMR than 1.0.

**Fig 2:**
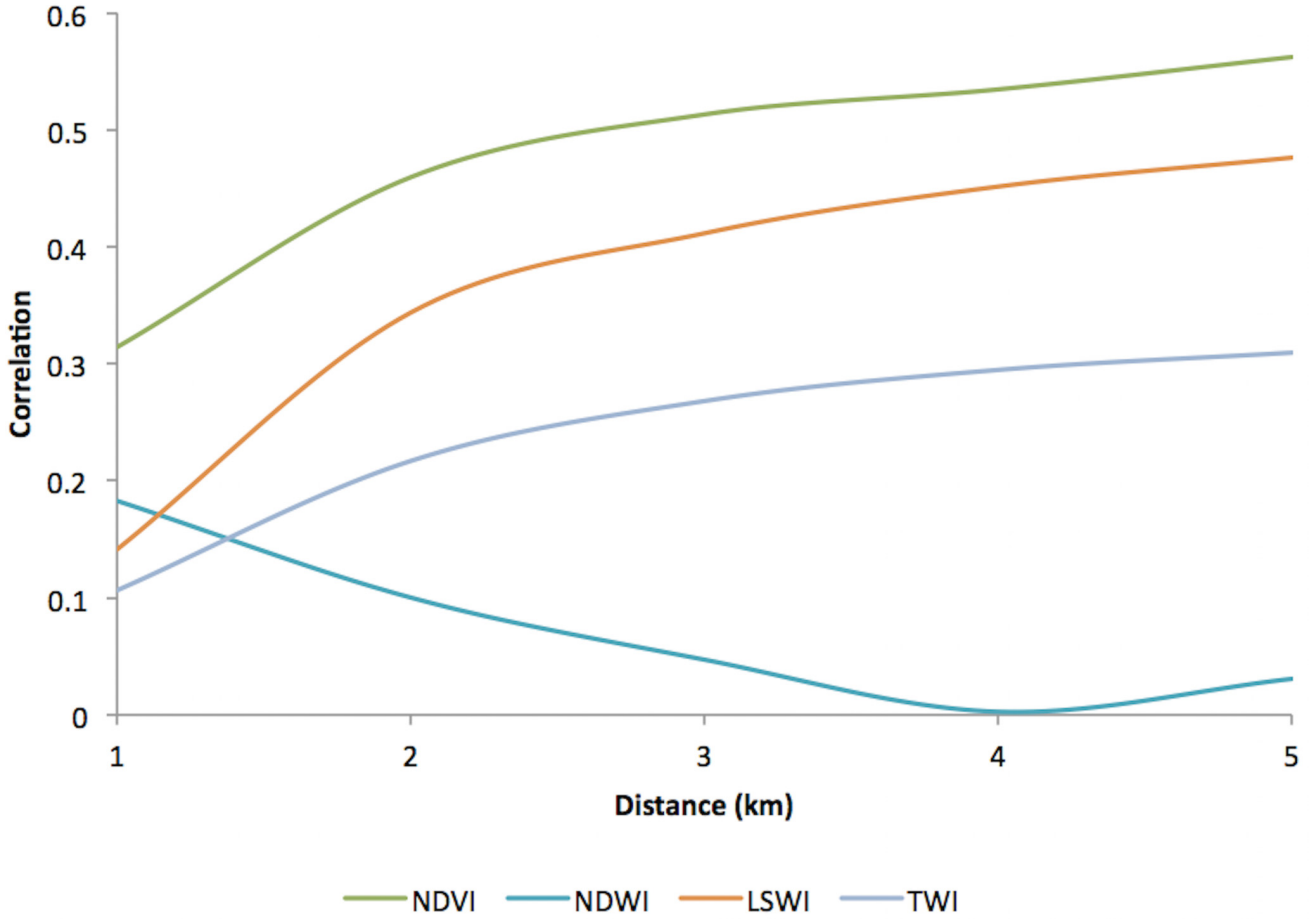
Absolute correlation values between environment-related covariates extracted from surrounding circular buffer from populated villages and EBSMR. Values were extracted from every 1 km distance circular buffer within 5 km (1, 2, 3, 4, and 5 km) from populated villages and then averaged to mean values. EBSMR, Standardized morbidity ratio estimated by empirical Bayese method; NDVI, Normalized difference vegetation Index; NDWI, Normalized difference water index; LSWI, Land surface difference index; TWI, Topographical wetness index.

**Fig 3:**
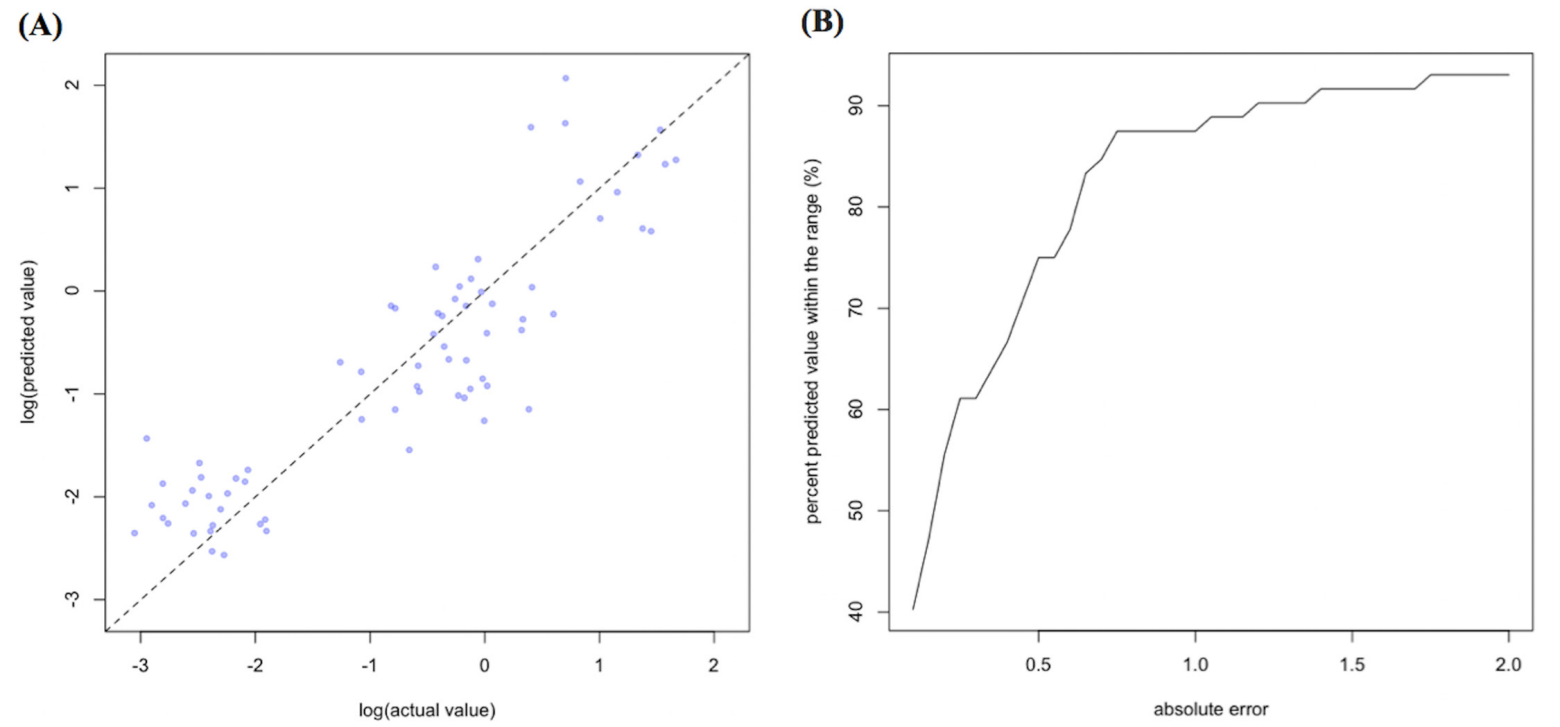
**The calibration plot (A) and proportions of predicted values within the range of absolute error (B) of the final model.** The dashed line in (A) represents 1:1 relationship of actual and predicted values.

**Fig 4:**
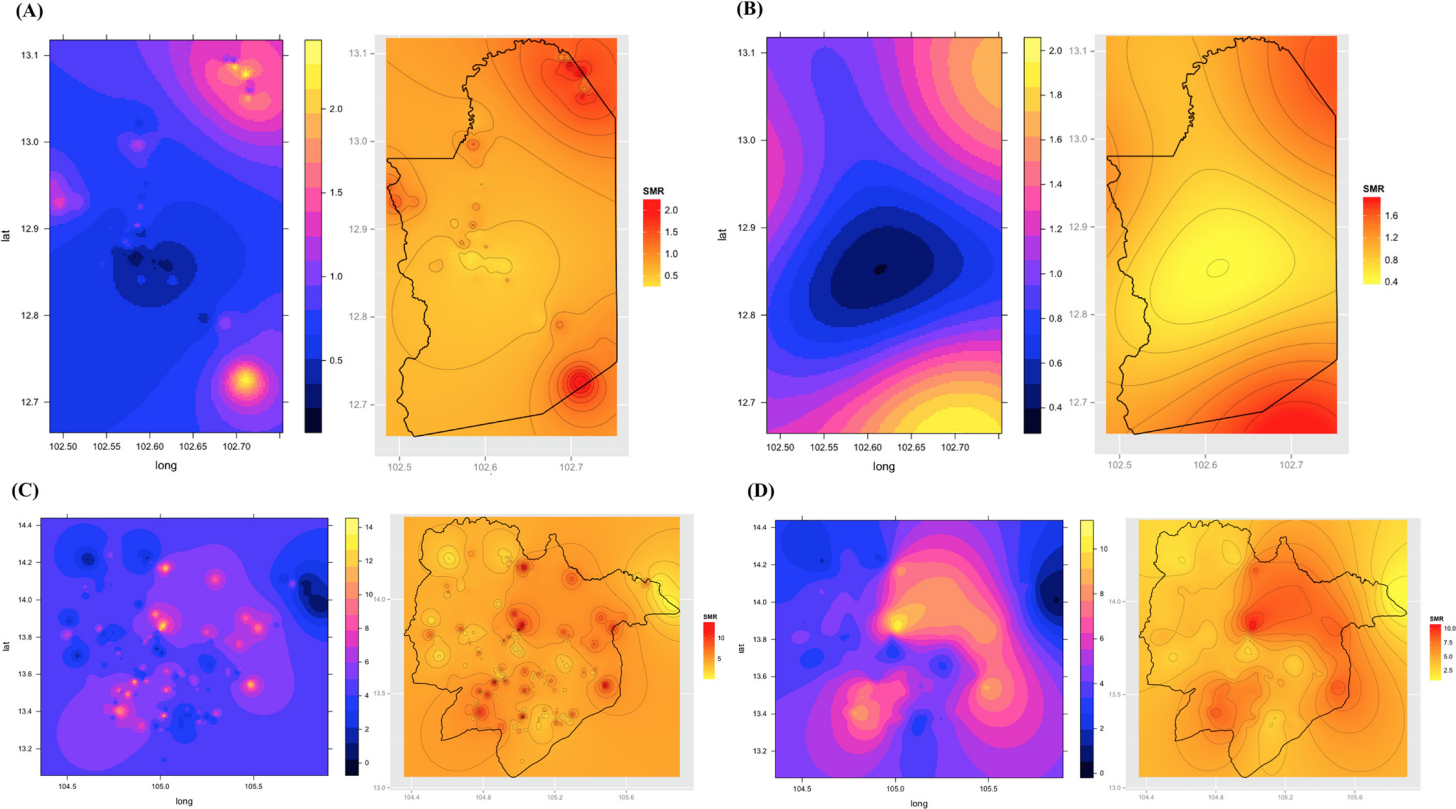
**Representative maps created using the proposed model for Pailin (A, B) and Preah Vihear (C, D) provinces in 2010.** Maps (A) and (C) are the risk maps created by the inverse distance weighed interpolation method (IDW). Maps (B) and (D) are the risk maps created by the ordinary kriging interpolation method. Maps on the right side of each figure are the risk map created by overlaying the political boundary of target area and the contour of estimated risk.

**Fig 5:**
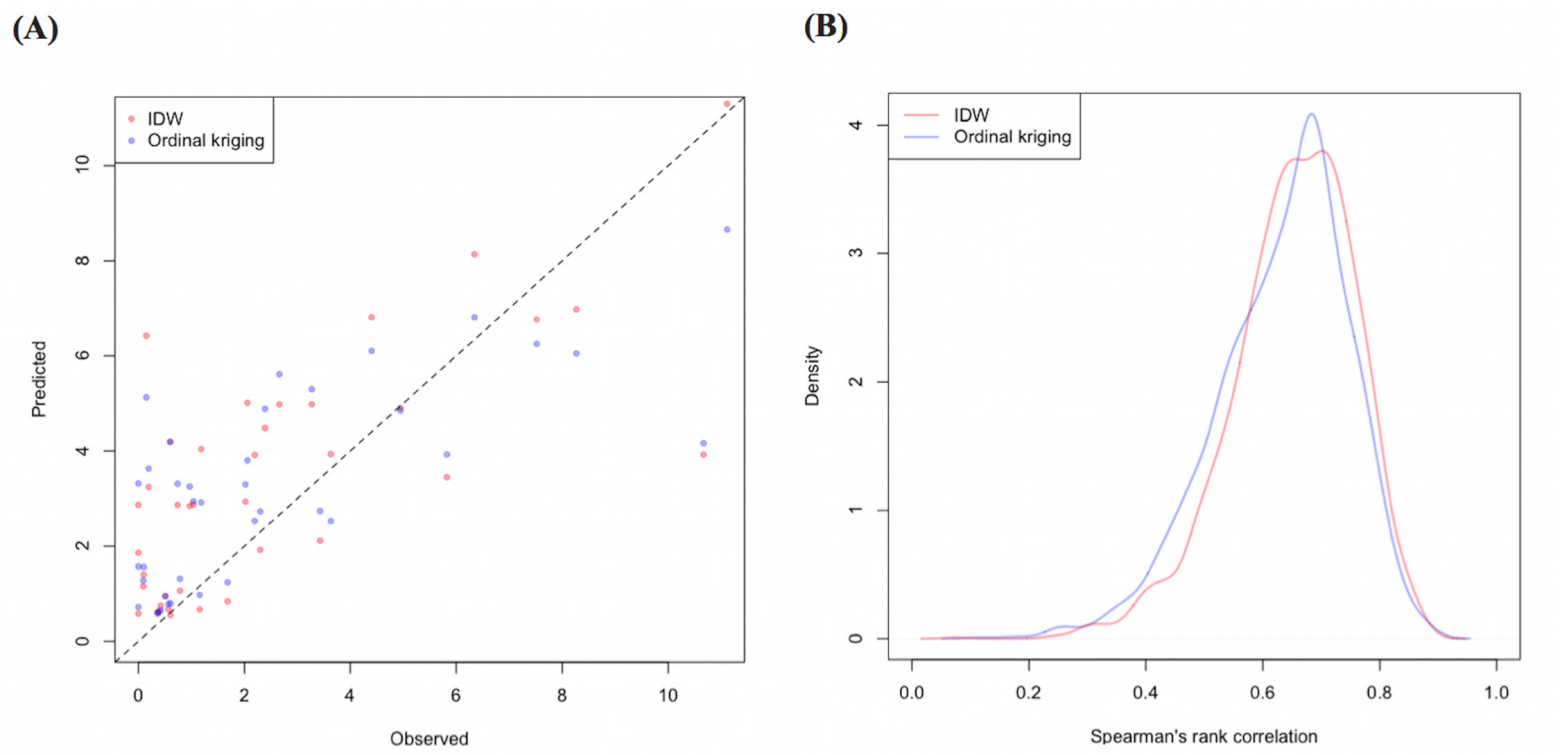
**Comparison of the standardized morbidity ratio calculated from geocoded case data with corresponding predicted values (A) and the kernel density plot of the resampled spearman’s rank correlation (B) in the risk map created by the model.** The dashed line in (A) represents 1:1 relationship of observed and predicted values. IDW, Inverse distance weighed method.

**Fig 6:**
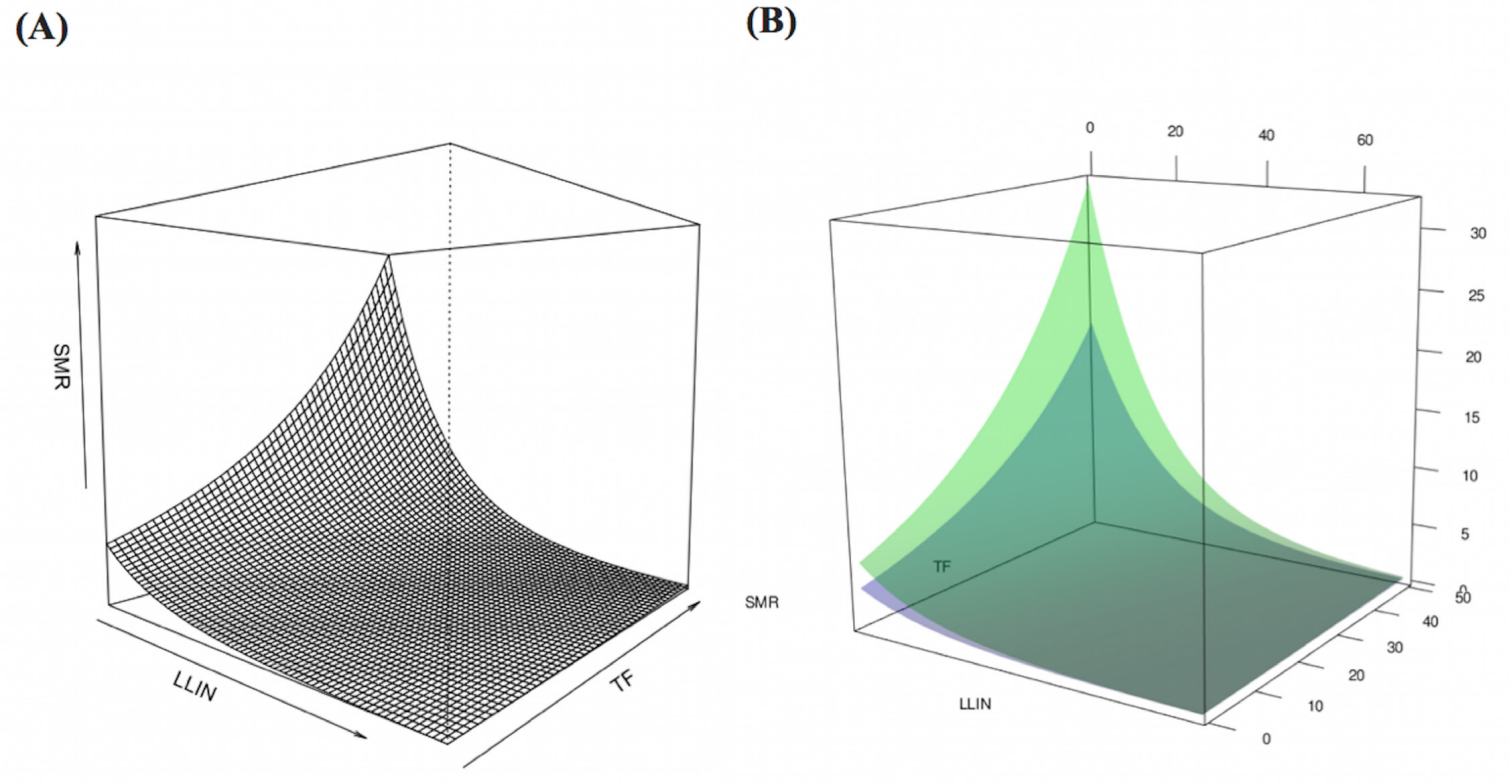
Computational simulations of expected the standardized morbidity ratio (SMR) under various conditions of LLIN coverage and treatment failure rate of artemisinin. (A) Relationship of two containment status indicators with expected SMR in Pailin Province. (B) Different patterns of expected outcomes from the combination of the two containment status indicators in the two provinces. The green surface corresponds to Pailin Province; the blue surface corresponds to Preah Vihear Province. LLIN, Long-lasting insecticide-treated net; TF, Treatment failure rate of artemisinin defined as test positive percentage on day 28 or day 42.

Of the 329,830 malaria cases reported in 2011–2013, 124,888 cases in 18 operational health districts in 10 western-Cambodian provinces were included in the analysis. The SMRs in each health operational district were smoothed using an empirical Bayes method. In contrast to the decreasing tendency of API in each district, estimated EBSMRs suggested remaining or even increasing tendencies of API in the endemic areas (Fig 1). Within 5 km of villages, the absolute correlation values between environmental variables (NDVI, LSWI, and TWI) extracted from surrounding circular buffers (from 1–5 km) and EBSMR were highest at 5 km and at 1 km for NDWI (Fig 2). Correspondingly, the Pearson correlation coefficient R^2^ of the model differed at each distance. Thus, the data collection ranges chosen for the model were 5 km for NDVI, LSWI, and TWI, and 1 km for NDWI. After selecting of the spatial covariates, the final model was used to estimate the SMR of each area (adjusted R^2^ = 0.774, Akaike information criterion AIC = 149.423). This model included NDVI, NDWI, TWI, *P*. *falciparum* temperature suitability index, LLIN_suf_, and TF_rate_. The parameter estimates for each variable are shown in Table 2. The calibration plot of the final model indicated good fitting of the predicted and actual values (Fig 3A), and the mean absolute error of this final model was 0.499. Fig 3B shows that 55.56% of predicted values were within the range of absolute error of ±0.2, 75% were in the range of ±0.5, and 87.5% were in the range of ±1. The estimated SMR for each village was calculated using the Bayesian modeling framework. Subsequently, fine-scale maps were created by the IDW method and ordinal kriging interpolation. The maps created from the predictive models for Pailin and Preah Vihear provinces are shown in Fig 4. Each map represents different risk representation patterns in accordance with interpolation method used. The map interpolated using the IDW method showed more spotted risk, which can help in identifying localized risky hotspots, whereas the map interpolated by ordinal kriging showed broader patterns, which provide a perspective of overall trends for optimizing healthcare resource distributions. Compared with geocoded case data, corresponding predicted values in this map showed conformity (Spearman’s rank correlation r = 0.662 with IDW and 0.645 with ordinal kriging; Welch’s t-test; Not significant), which showed that the cross-scale predictions corresponded well with the actual case reports (Fig 5A). The 95% confidence intervals for the IDW and ordinal kriging methods were 0.414–0.827 and 0.368–0.813, respectively, showing a steep peak in the kernel density plot at around 0.65–0.7 (Fig 5B). The visual representations of hotspots in the fine-scale map created here confirmed that they were aligned with actual areas at high risk, which were identified by other sources [36, 48, 54], through visual assessments by a number of healthcare providers and experts in the geographic information system. Thus, using this model, expected outcomes under given conditions of LLIN_suf_ and TF_rate_ were simulated. The visual representations demonstrated the different patterns of expected outcomes from the combination of these two containment status indicators in respective areas (Fig 6).

**Table 2:** Parameter estimates selected for the final generalized linear regression model.

Category	Variable	Parameter estimate	Standard error	P-value
Vegetation	NDVI (5 km)	7.446	1.947	<0.001
Water	NDWI (1 km)	-24.330	5.009	<0.001
Geography	TWI (5km)	-1.707	0.6346	0.009
Temperature	*P*. *falciparum* Temperature suitability index (*Pf*TSI)	0.0002681	0.0000403	<0.001
Vector control	Sufficient ownership of LLIN ^a^	-0.06387	0.007157	<0.001
Treatment	Treatment failure rate by artemisinin combination Therapy ^b^	0.03611	0.008309	<0.001

^a^ Proportion of household in which distributed mosquito net covers 2 persons or less per net.

^b^ Test positive for *P*. *falciparum* on day 28 or day 42

NDWI, Normalized difference water index; NDVI, Normalized difference vegetation index; TWI, Topographical wetness index; LLIN, Long lasting insecticide-treated net

## Discussions

As the malaria elimination effort progresses, it has become increasingly important to identify the residual foci of malaria transmission to address the remaining challenges of preventing residual transmissions and preventing the emerging artemisinin-resistant malaria from spreading to protect immunologically susceptible populations. The fine-scale maps that we have created will enable more focused containment actions, such as targeted surveillance, preventive measures, and monitoring for treatment failure, which require intensive support for local health practitioners. A previous report suggested that remarkable proportions of patients in western Cambodia still had malaria parasitemia on day 3 after starting artemisinin combination therapy, although symptom resolutions were observed within this period [11]. Thus, treatment monitoring is important for preventing patients from discontinuing treatment and developing drug resistance. Interestingly, the visual representations of our maps were similar to those of the Malaria Atlas Project; however, our maps displayed a finer level of risk distributions. Some of the differences between the two sets of maps can be explained partially by spatial and temporal variations in the source data. The comparison of predicted risk with geocoded case data confirmed that the areas predicted to be at high risk were likely the areas where the appropriate level of attentions and support are needed. Areas predicted to be at high risk can be distinguished easily from other regions at low risk, which will provide information to quantify expected outcomes from a combination of containment status indicators. These results suggested that these fine-scale maps can play important roles in current situations in Cambodia.

We also describe an application of SMR using API reported in routine aggregated surveillance data to quantify the spatial distribution of risk by capturing the environmental context and containment status indicators in the model under low-to-moderate transmission settings. We found that the remaining or even increasing tendency of SMR reflected the relative risk of malaria in the studied areas during the research period, which can be a useful measure for deciding the allocations of limited healthcare resources. Sturrock et al. [33] built a prediction model using routine aggregated case data and created a fine-scale risk map for Swaziland. In their model, mean temperature and travel time to health facilities were the predictors of both the pixel scale and the coarser district scale of risks. Lowe et al. [42] reported various kinds of predictors such as altitude, living conditions, urbanization, precipitation, and temperature. The variables that we chose for our model were similar in terms of using environmental and human behavior-related variables for malaria risk predictions. Although altitude may be related to malaria ecology, we did not incorporate this variable into our model. Nevertheless, the risk was well explained, probably because of the relatively flat terrain in most of the area that we studied. Of note, the data collection distances from each village for environment-related covariates affected the risk predictions made by the model. The distances selected for the model development were different for NDVI and NDWI, which partially reflects human interactions with the living conditions that exist around human communities. The relationships between *Anopheles* mosquito numbers that cause malaria transmission and distance from mosquito bleeding sites have been reported previously [60–62]. According to surveillance reports [41, 63–64], malaria prevalence decreased by distance from forests. The relationship with the distance from environmental features for malaria risk modeling, such as the proximity of water puddles [65] and health facilities [34], have been considered. The effect of distance for the vegetation and water indices used in our study indicates such environmental features are interrelated with human communities in different ways. Forest workers often work in forests that are several kilometers away from the communities in which they live, whereas the activity ranges of vectors are limited to short distances from their breeding habitat. The maps created in this study suggest that the spatial heterogeneity of disease risk can be explained by such environmental context disparities. Our approach shows that distance from living communities can be a useful reference in which to consider environmental context disparities for cross-scale prediction of disease risk on a fine-scale. The relative risk specified from the surrounding environmental context can be described over a wide area, while maintaining the uniformity of unknown conditions, using remote sensing data from space satellites.

It is desirable to use micro data, such as household level data, to build fine-scale risk maps. However, this kind of micro data is often inaccessible and hence they cannot be used for mapping. The encouraging results that we obtained for fine-scale risk prediction in the modeling framework enabled the size of the effect to be visualized from different combinations of containment status indicators. The simulation results demonstrated that the predicted outcomes were different under each environmental context, which provides an opportunity for evaluating interventions considering environmental situations in target areas. Moreover, expected interventional outcomes can be mapped, allowing decision-makers to assess different combinations of interventional approaches considering several constraints such as detailed population characteristics, specific local issues, and resource constraints in a target area. Generally, the reliability of data is a critical factor for creating relevant models to be used in the real world. Under low transmission settings, passive surveillance systems have difficulty in capturing enough reliable case numbers to reflect the actual situations [66]. Although variations in the reliability of data reported from each area are likely to exist, the mapping approach described here can add more reciprocity among stakeholders than simply recording aggregated case numbers, which will encourage more effective report-and-utilization cycles and provide an opportunity for effective data utilization.

While our approach generated several supportive results in terms of fine-scale risk predictions under a low-to-moderate transmission setting, several important limitations and considerations for future work should be considered. First, containment status indicators other than LLIN_suf_ and TF_rate_ were not considered in the present model. The expected outcomes of interventional efforts could be obtained from the results of various activities, which may not be explained by a simple additive effect, but rather through the interaction of these activities. In our model, we considered the interaction between LLIN_rate_ and TF_rate_, but the result did not improve. Therefore, interactions to describe the complex realty should be considered for practical applications for assessing the effectiveness of interventions. Second, the influence of migrant populations and time series variations of the risk were not considered in the modeling framework. The dynamics of human carriers that drive parasite transportation between regions can be quantified using spatially explicit mobile phone data and malaria prevalence information [67]. By incorporating these factors into the modeling framework more useful models could be developed. Because we used API to calculate the SMR, the environment-related predictor variables were reduced to yearly average. For the spatial granularity of data, deciding appropriateness of the time granularity is a perplexing issue because of difficulties in detecting adequate case numbers for reliable risk modeling from micro data. The appropriateness for deciding the region of interest for data collection is also difficult to determine because calculations of the denominator (i.e. prevalence of reference population) of the SMR are influenced by this factor. Finally, the treatment seeking behavior varies spatially, which may affect the reporting bias of case data. Sturrock et al. addressed this issue in their modeling approach using Swaziland malaria information system [33]. Unfortunately, this kind of information in Cambodia was not available from publicly available sources. Thus, we need to conduct field survey in the sampled place of target area if this aspect needs to be incorporated. However, in addition to the case reported from public facilities, cases reported from village malaria workers providing primary healthcare services to the community were also counted in the surveillance report we used. Since the village malaria worker program is active in northwest Cambodia, this structure can improve the coverage of potentially detectable cases to a certain extent. One of the strengths of our approach is that the maps were created mostly from public available data. Therefore, map authors need to collect complementary data from the field if it is necessary considering the balance of timeliness and reliability of the map.

Like all programs, malaria elimination action programs need specific plans with realistic time limits and well-defined parasitological and entomological goals [35]. Maps created by the modeling framework developed here can provide opportunities for establishing realistic goals using current resources. Furthermore, the maps can provide useful information both quantitatively and qualitatively for monitoring and evaluating interventional activities, while providing decision-makers with a platform for cross-scale wandering to help make decisions for efficient healthcare resource use. Our approach is simply a quantitative prediction technique for using existing dataset, and thus can only play a part in the whole healthcare information system for malaria elimination. Clearly, the divergences of the prediction from a real world situation need to be considered. Nevertheless, the adjustments in malaria quantification contribute further steps in a system that is working toward malaria elimination.

## Conclusions

Using routine aggregated surveillance reports combined with environmental data and non-environmental anthropogenic data, regional malaria risks can be well explained with the approach described here. The modeling framework was used to created fine-scale risk maps under the low-to-moderate transmission setting where reinvestigations of existing risk modeling approaches were needed. We have demonstrated a mathematical modeling approach for SMR using API from routine aggregated surveillance report and generated cross-scale predictions within a modeling framework that correspond to environmental context disparities to create malaria risk maps on a fine-scale. Different representations of simulated outcomes from containment status indicators can provide useful insights for tailored planning of action alternatives considering regional malaria endemicity.

## Supporting Information

S1 Fig Schematic overview of modeling and mapping method for the fine-scale malaria risk map. (DOCX)
