# Supplementary material for: Fine-Scale Mapping by Spatial Risk Distribution Modeling for Regional Malaria Endemicity and Its Implications under the Low-to-Moderate Transmission Setting in Western Cambodia
Source: PLoS One. 2016 Jul 14;11(7):e0158737. doi: 10.1371/journal.pone.0158737 (PMC4944927; doi:10.1371/journal.pone.0158737)
Supplement: S1 Fig — (DOCX) [file pone.0158737.s001.docx]

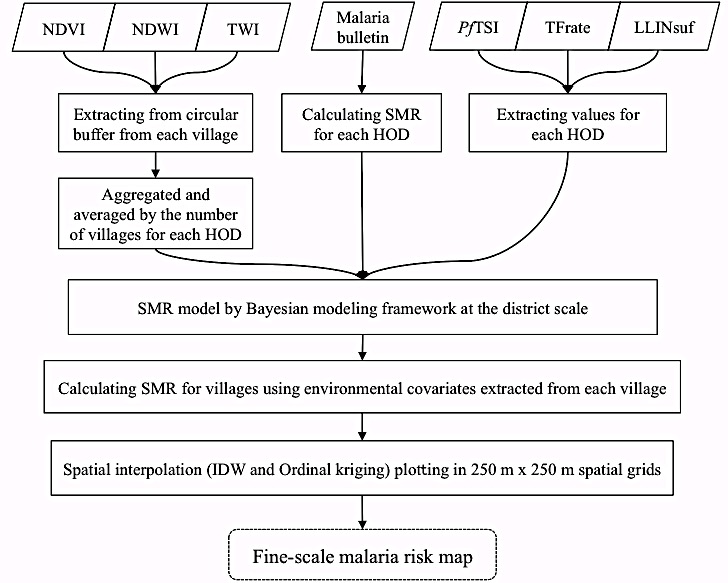


**S1 Fig. Schematic overview of modeling and mapping method for the fine-scale malaria risk map**

NDWI, Normalized difference water index; NDVI, Normalized difference vegetation index;

TWI, Topographical wetness index; *Pf*TSI *, P. falciparum* Temperature suitability index;

LLINsuf, Sufficient ownership of long lasting insecticide-treated net; HOD, Health operational district;

TFrate, Treatment failure rate (Test positive for *P. falciparum* on day 28 or day 42);

SMR, Standardized morbidity ratio; IDW, Inverse distance weighed method
